# P-913. Etiologies, Clinical Characteristics and Prognostic impact of Increased Intracranial Pressure in Adults with Encephalitis

**DOI:** 10.1093/ofid/ofae631.1104

**Published:** 2025-01-29

**Authors:** Hazim Allos, Ashley N Heck, Paris Bean, Rajesh Gupta, Ralph Habis, Romergryko Geocadin, John Probasco, Arun Venkatesan, Rodrigo Hasbun

**Affiliations:** UTHealth Science Center at Houston, Houston, Texas; McGovern Medical School, UTHealth Science Center, Houston, TX, Houston, Texas; UTHealth Science Center at Houston, Houston, Texas; UTHealth Science Center at Houston, Houston, Texas; Johns Hopkins University School of Medicine, Baltimore, Maryland; Johns Hopkins University School of Medicine, Baltimore, Maryland; Johns Hopkins University School of Medicine, Baltimore, Maryland; Johns Hopkins University School of Medicine, Baltimore, Maryland; UT Health Mc Govern Medical School, Houston, Texas

## Abstract

**Background:**

Encephalitis involves symptoms like reduced consciousness and seizures, diagnosed via lumbar puncture and brain imaging. Increased intracranial pressure (ICP) and its effect is well described in meningitis/ventriculitis and serve as poor prognostic factors. Data on ICP in encephalitis is limited, highlighting the need for further research.

**Table 1.**
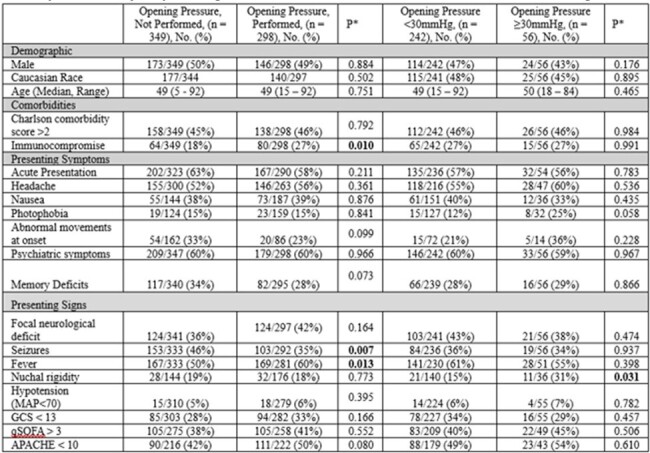
Demographic, Clinical Characteristics, and Initial Presentations of 647 Adults with Encephalitis by Opening Pressure Measurement Status and Degree Abbreviations: APACHE, Acute Physiology and Chronic Health Evaluation; GCS, Glasgow Coma Scale; MAP, Mean Arterial Pressure; qSOFA, Quick Sequential Organ Failure Assessment.

**Methods:**

Retrospective study at University of Texas and Johns Hopkins, 2002-2023, on 647 patients over 17 years old with encephalitis. Data analyzed via SPSS software.

**Table 2.**
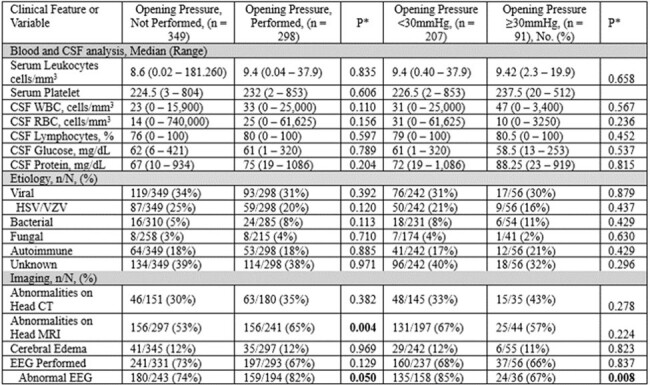
Laboratory Findings, Etiology, and Imaging Results in Encephalitis Patients by Opening Pressure Measurement Status and Degree Abbreviations: CSF, cerebral spinal fluid; CT, computed tomography; EEG, electroencephalogram; HSV, herpes simplex virus; MRI, magnetic resonance imaging; PMN, polymorphonuclear leukocyte; RBC, red blood cell; VZV, varicella zoster virus; WBC, white blood cell.

**Results:**

Our study had 647 patients, with ICP measured in 298 (46%). Patients were categorized by ICP levels: < 30mmHg (242) and ≥30mmHg (56). Demographics such as gender, race, and age showed no variance across ICP. Comorbidity profiles were similar, though immunocompromised patients more frequently had ICP measurement (p=0.010). Symptoms like photophobia were uniformly distributed, but seizures and fever were more common in those measured for ICP (p=0.007 & p=0.013). Nuchal rigidity was higher in those with ICP ≥30mmHg (31% vs. 15%, p=0.031).

Blood and cerebrospinal fluid analyses did not differ across groups. The leading etiologies identified were viral (32.9%) and autoimmune (18.1%), with no cause specifically linked to elevated ICP. Neuroimaging was more likely to be abnormal in patients with ICP measured (65% vs. 53%, p=0.004). Those with abnormal EEGs were more likely to have ICP measured (82% vs. 74%, p=0.050), but abnormal EEGs were less common in patients with ICP >30mmHg (p=0.008).

No differences were found in given treatments like vancomycin, acyclovir, or steroids between ICP groups. ICU admission, vasopressor support, and ventilation needs were comparable, though patients with higher ICP had longer median hospital stays (12 days vs. 10 days, p=0.012) and mechanical ventilation (1.5 days vs. 0 days, p=0.005). Mortality and readmission rates showed no differences, but patients with higher ICP had worse Glasgow Outcome Scale (GOS) scores (61% vs. 46%, p=0.045).

**Table 3.**
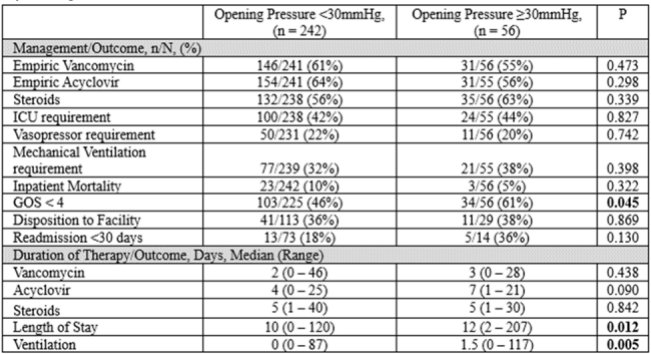
Management Approaches and Outcomes in Encephalitis Patients Categorized by Opening Pressure Levels Abbreviations: ICU, intensive care unit; GOS, Glasgow outcome scale.

**Conclusion:**

This retrospective study on ICP in encephalitis across two centers emphasizes presentation variability and the need for standardized diagnostic and management protocols. Noted differences include ventilation duration, hospital stays, and GOS in patients with higher ICP.

**Figure 1.**
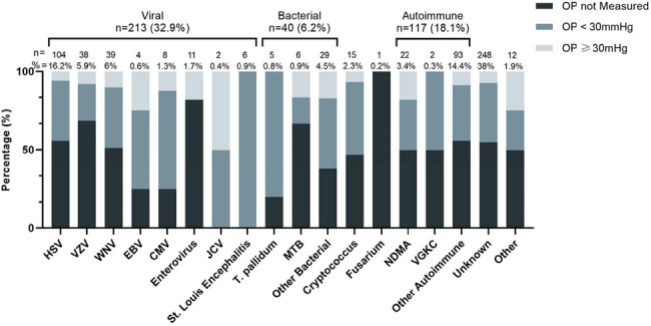
Breakdown of 647 cases of encephalitis, categorized by their determined etiologies and ICP measurements. The chart illustrates the distribution across three groups: OP not measured, OP <30mmHg, and OP ≥30mmHg, detailing the total number of cases alongside the percentages for each etiological category, including viral, bacterial, autoimmune, and unknown origins. CMV; Cytomegalovirus, EBV; Epstein-Barr Virus, HSV; Herpes Simplex Virus, JCV; JC Virus, MTB; Mycobacterium tuberculosis NMDA; N-Methyl-D-Aspartate, T. pallidum; Treponema pallidum, VGKC; Voltage-Gated Potassium Channel, WNV; West Nile Virus.

**Disclosures:**

**John Probasco, MD**, Genentech: Study site investigator for multicenter study **Rodrigo Hasbun, MD MPH FIDSA**, Biomeriaux: Grant/Research Support|Biomeriaux: Honoraria

